# Exploring the latent structure of behavior using the Human Connectome Project’s data

**DOI:** 10.1038/s41598-022-27101-1

**Published:** 2023-01-13

**Authors:** Mikkel Schöttner, Thomas A. W. Bolton, Jagruti Patel, Anjali Tarun Nahálka, Sandra Vieira, Patric Hagmann

**Affiliations:** 1 Connectomics Lab, Department of Radiology, Lausanne University Hospital and University of Lausanne (CHUV-UNIL), Lausanne, Switzerland; 2 Neurosurgery Service and Gamma Knife Center, Lausanne University Hospital (CHUV), 1011 Lausanne, Switzerland; 3 Department of Psychosis Studies, Institute of Psychiatry, Psychology and Neuroscience, King’s College London, London, UK

**Keywords:** Human behaviour, Neuroscience

## Abstract

How behavior arises from brain physiology has been one central topic of investigation in neuroscience. Considering the recent interest in predicting behavior from brain imaging using open datasets, there is the need for a principled approach to the categorization of behavioral variables. However, this is not trivial, as the definitions of psychological constructs and their relationships—their ontology—are not always clear. Here, we propose to use exploratory factor analysis (EFA) as a data-driven approach to find robust and interpretable domains of behavior in the Human Connectome Project (HCP) dataset. Additionally, we explore the clustering of behavioral variables using consensus clustering. We find that four and five factors offer the best description of the data, a result corroborated by the consensus clustering. In the four-factor solution, factors for Mental Health, Cognition, Processing Speed, and Substance Use arise. With five factors, Mental Health splits into Well-Being and Internalizing. Clustering results show a similar pattern, with clusters for Cognition, Processing Speed, Positive Affect, Negative Affect, and Substance Use. The factor structure is replicated in an independent dataset using confirmatory factor analysis (CFA). We discuss how the content of the factors fits with previous conceptualizations of general behavioral domains.

## Introduction

One of the main goals of cognitive neuroscience is to map mental processes to brain physiology. However, looking at the literature, a one-to-one mapping is rarely, if ever, achieved. This failure to map mental functions to brain structures or activity may partly be due to how psychological constructs are ill-defined or unrelated to the way the brain encodes information^1^, which may also contribute to the low replicability of psychology studies^2^. There is therefore the need for a *cognitive* *ontology*—a systematic description of mental capacities and their relationships, derived using data-driven methods^3^. Existing ontologies derived from expert knowledge such as the Research Domain Criteria (RDoC^4^) and Diagnostic and Statistical Manual of Mental Disorders (DSM^5^) have been shown to fit neuroimaging results less efficiently than data-driven ontologies^6^.

Recently, interest has shifted from finding direct mappings between brain physiology and behavior to focusing on predictive modeling of behavior using brain imaging data, with the aim to develop robust biomarkers of behavior^7,8^. Evidently, achieving this requires high-quality behavioral measures^9^. The Human Connectome Project (HCP^10^), which has publicly released a compilation of behavioral and neuroimaging data for a large (*N* > 1000) sample of healthy subjects, has been a popular resource to pursue this goal^11–13^. A necessary condition is the availability of robust and interpretable summary measures of behavior. In this study, we propose to use exploratory factor analysis (EFA) to derive meaningful behavioral domains that summarize the variables of the HCP data set. Additionally, we show that the factor structure found in this way is mirrored in a discrete clustering. We go on to establish how well these factors replicate in independent data using the same variables, as well as on a completely different dataset, collected by the UCLA Consortium for Neuropsychiatric Phenomics^14^.

EFA has been successfully deployed to this aim on other large-scale datasets^15,16^. Others have used EFA on a subset of the HCP behavioral variables to derive a general intelligence factor^7^. In factor analysis the goal is to find latent variables that are viewed as causes of the measured variables. This cause-and-effect structure is why we believe that EFA is a suitable method to derive a data-driven ontology, compared to other dimensionality reduction techniques that have a different theoretical foundation. Given that the factor model also includes a noise term that captures variance specific to each measure, it should deliver a robust latent factor solution.

To our knowledge, this is the first study to use EFA to explore the latent structure of behavior in the HCP data. We show that a description of behavior with four to five factors provides an optimal trade-off between robustness, accuracy and interpretability of the solution, and we discuss the potential of the unraveled latent structure to reveal the neurobiological substrates of behavior in follow-up work.

## Methods

### Data

This study used the behavioral data of the HCP Young Adult 1200 Subjects release^10^. Participants gave informed consent, and all recruitment and acquisition methods were approved by the Washington University Institutional Review Board (IRB), following all relevant guidelines and regulations. The dataset consisted of 1206 subjects (656 female) with a median age of 29 years (*min* = 22, *max* = 37). Subjects were pseudo-randomly split equally into a discovery and a replication data set, to be used for the exploratory and confirmatory factor analyses, respectively. To ensure independence of the two subsets, families were kept intact by assigning siblings always to the same data set. The discovery set subsequently consisted of 602 subjects (328 female) with a median age of 29 years (*min* = 22, *max* = 36), and the replication set comprised 604 subjects (328 female) with a median age of 29 years (*min* = 22, *max* = 37).

The input variables for the factor analysis were chosen based on the following criteria: they had to be numerical, include less than 10% missing values (following the criteria by^17^), and should have at least one correlation coefficient with small effect size or higher (r > 0.1^18^). Variables also should not be composite measures of other items in the data set to avoid biases resulting from the same information being entered in the analysis twice. For variables with an unadjusted version and one adjusted for age and/or gender, the unadjusted variant was used. Following these criteria, 87 variables from the HCP sections Alertness, Cognition, Emotion, Motor, Personality, Sensory, Psychiatric and Life Function, Substance Use, and Scanner Tasks were used for further analysis. A more detailed documentation on the variables’ choice can be found in a dedicated notebook in the Github repository.

To demonstrate replicability on completely independent data, we used a dataset from the UCLA Consortium for Neuropsychiatric Phenomics^14^, which included 272 subjects (117 female) with a median age of 31 (*min* = 21, *max* = 50). Some of the subjects were diagnosed with a mental disorder, with 49 subjects fulfilling the criteria for bipolar disorder, 43 for ADHD, 39 for schizophrenia, and 11 for schizo-affective disorder; 130 subjects were healthy controls. See the dataset reference^14^ for more specific information on the sample. Following the same criteria as for the HCP data, we chose 96 variables that went into the analysis.

### Preprocessing

Discovery and replication data from the HCP dataset, as well as the UCLA data were preprocessed independently with the following procedure. Missing values were imputed using Multiple Imputation by Chained Equations (MICE^19^). We regressed out age and gender by entering them as predictors in a linear regression and proceeding with data analysis using the residuals (difference between real and predicted values). Additionally, reaction times and other error measures were inverted (see the variables’ choice notebook in the Github repository). Finally, data were z-scored across subjects.

### Exploratory factor analysis

We used hierarchical factor analysis^20^ using maximum likelihood (ML) estimation with orthogonal (varimax) rotation on the discovery data to explore the latent variable structure, using the “Factor Analyzer” package implemented in Python^21^. The same estimation was also used on the replication data set for comparison, as well as for the UCLA data. For the replication data, the same numbers of factors as established in the discovery set were used. In the UCLA dataset, we chose the number of factors that corresponded best to the level of granularity of the factor solutions of the HCP dataset. The factor model describes each variable as a weighted sum of factor values plus a noise term, according to

1 $${x}_{i,v}\approx {\sum }_{f=1}^{k}{l}_{v,f}{F}_{f,i}+{\upepsilon }_{\mathrm{i},v}$$

where *x*_*i,v*_ is the value of subject *i* on variable *v*, *l*_*v,f*_ is the loading of variable *v* on factor *f*, *F*_*f,i*_ is the factor score of subject *i* for factor *f*, *k* is the number of factors, and ${\upepsilon }_{\mathrm{i},v}$ is a Gaussian error term^22^. In other words, we assume an underlying factor structure yielding shared variance across variables, as well as the additional presence of variable-specific variance and noise.

In practice, the covariance matrix of the data is thus approximated as a product of factors (shared variance), plus a *uniqueness* *matrix* (variable-specific variance), given by

2 $$\boldsymbol{\Sigma }\approx \mathbf{L}\mathbf{L}\mathbf{^{\prime}}+{\mathbf{U}}^{2}$$

where ***Σ*** is the reconstructed covariance matrix, **L** is the matrix of loadings, and **U**^**2**^ is the matrix of uniquenesses—a diagonal matrix whose values can be interpreted as the amount of variance unique to each variable. The goal is to make the reconstructed covariance matrix ***Σ*** as similar to the sample covariance matrix **S** as possible, according to some error *E*. For maximum likelihood estimation this error is given by the equation

3 $$\mathrm{E}=\frac{1}{2}\mathrm{tr}({(\mathbf{S}-\boldsymbol{\Sigma }){\boldsymbol{\Sigma }}^{-1})}^{2}$$

The squared factor loadings over all variables for a given factor quantify the variance that it explains:

4 $$E{V}_{f}={\sum }_{v}{l}_{v,f}^{2}$$

Parallel analysis^23^ was used to identify the maximum number of factors to be considered, similar to^16^. For this, factor solutions were computed for every number of factors from one to the number of variables. Synthetic data were created by filling a matrix with the same dimensions as the original one (602 subjects, 86 variables) with random values drawn from a normal distribution (µ = 0, σ = 1). This was repeated 20 times, fitting the factor model and saving the squared factor loadings for each iteration. The averages per factor across iterations from the synthetic data (which quantify shared variance when there are no underlying dependences between variables) were then compared to their equivalents from the real data. The crossover point where the average squared loadings of the synthetic data become greater than the squared loadings of the real data defined the maximum number of factors considered for closer evaluation.

All possible numbers of factors from 1 to this maximum number were then examined with regard to the criteria of (a) *model* *fit*, (b) *interpretability*, and (c) *robustness*. *Model* *fit* was measured as the percentage of total variance explained and should be as high as possible while still maintaining the other two criteria. Regarding *interpretability*, we follow the guidelines by^24^, suggesting that each factor should have at least three variables with loadings > 0.4.

*Robustness* was assessed using the following subsampling procedure: for 1000 iterations, a sample of 80% of subjects of the discovery set was drawn. Factors were extracted and rotated as described above for each number of factors to consider, saving all loadings. After running all 1000 folds, the factors were reordered such that the order is the same over all samples. To this end, the Hungarian algorithm^25^ was applied with one minus the absolute correlation matrix as the cost matrix. Finally, the averaged absolute pair-wise correlation of each factor over samples was considered as a measure of robustness.

To create a hierarchical representation, we calculated factor scores for each subject using Bartlett’s method^26^, a univocal and unbiased approach. Factor scores offer an estimate of where a subject falls on the latent dimension described by the factor. The scores were then correlated between consecutive numbers of factors (factor one of the one-factor solution to factors one and two of the two-factor solution and so on). Each number of factors now constituted a level in the hierarchy, while the correlation coefficients can be interpreted as the “flow” of the variance across levels^20^.

Factor loadings were correlated between discovery and replication datasets to quantify the similarity of factors between the two sets. For this, only the numbers of factors that emerged as optimal from the previous analyses were considered.

### Consensus clustering

To verify the results from EFA and explore latent divisions that might not show in terms of dimensions, but clusters, we utilized consensus clustering^27^. The same procedure was followed on the discovery and replication sets of the HCP data. The method leverages a subsampling procedure to find the consensus over multiple runs of a clustering algorithm. For every pair of items, a *consensus* *index* is calculated, which quantifies the fraction of times the items were clustered together out of the times they were drawn in the same subsample.

We employed *K*-means clustering as implemented in Scikit-Learn^28^ on a subsample of 80% of variables for 1000 iterations for each respective sub-dataset. For every iteration, the algorithm was initialized with 10 different centroid seeds. Clustering from *K* = 2 up to the maximum number of factors considered in EFA was performed. To assess the quality of the clusters, the *cluster* *consensus* was calculated as the average consensus index for all variables in each cluster.

Additionally, to assess how similar cluster solutions were across subsamples, a *consensus* *score* was developed. For this, all consensus indices were binned into values below and above chance level. An average for both bins was computed, and the difference between these averages was taken as the consensus score.

In more details, the chance level for a given clustering solution is $\frac{1}{K}$, where *K* is the number of clusters. The average consensus index for each bin is then

5 $$above= \frac{\sum {ci}_{above}}{{n}_{above}}; below= \frac{\sum {ci}_{below}}{{n}_{below}}$$

where *ci*_*above*_ (*ci*_*below*_) are the consensus indices above (below) chance level, and *n*_*above*_ (*n*_*below*_) is the number of consensus indices above (below) chance level. The consensus score *CS* is then calculated as

6 $$CS=above - below$$

The reasoning is that if there is perfect consensus, variables will either always be in the same cluster (having a consensus index of 1), or never (consensus index of 0). The consensus index indicates the fraction of samples in which a pair of items is clustered together over all subsamples, normalized by how many times the items were in the same sample^27^. Conversely, when there is no consensus, all pairs of items will be clustered together by chance, which depends on how many clusters were chosen. Consequently, all consensus indices will then be scattered around chance level and the difference between the averages of each bin will be close to zero. The range of the consensus score is thus between (near) zero for no consensus and one for perfect consensus.

After deciding on the number of clusters, we used *K*-means clustering on the consensus matrix to extract the final cluster labels for both the discovery and the replication data sets. To compare the similarity of the clustering between the two sub-datasets, the adjusted rand index^29^ and the adjusted mutual information^30^ were calculated using Scikit-Learn^28^.

### Confirmatory factor analysis

Confirmatory factor analysis (CFA) with maximum likelihood estimation as implemented in Lavaan^31^ was employed to test the latent structure found in the previous step in the replication data set. Based on the results from EFA, the models to be tested were the 4-factor model and the 5-factor model. Following the recommendations by^32^, variables with absolute loadings > 0.45 were used as the indicator variables for each factor, meaning the variables that were allowed to have non-zero loadings in the CFA. Effectively, only those variables contributed to model evaluation. The loadings for these variables were thus estimated freely, all others were set to 0, and variances of factors fixed to a value of 1. Additionally, a model that was based on the HCP categories as specified in the HCP data handbook was entered into the analysis. The following fit indices were computed for each model: Comparative Fit Index (CFI), Tucker-Lewis Index (TLI), Root Mean Square Error of Approximation (RMSEA), and Standardized Root Mean Square Residual (SRMR). The CFI compares the fit of the model to a baseline null model with uncorrelated variables, and the TLI additionally corrects for model complexity. Both indices take values between 0 and 1, with larger values reflecting better fitting, and are *relative* fit measures. In contrast, the RMSEA and SRMR are *absolute* fit measures that function without comparison to a baseline model. The RMSEA quantifies the discrepancy between the hypothesized model and the population covariance matrix, while the SRMR is the square root of the discrepancy between the sample covariance matrix and model covariance matrix. Both range from 0 to 1, with lower values denoting better fitting. Here, the RMSEA includes parsimony correction^33^.

## Results

### Dimensionality of the factor space

**Figure 1:**
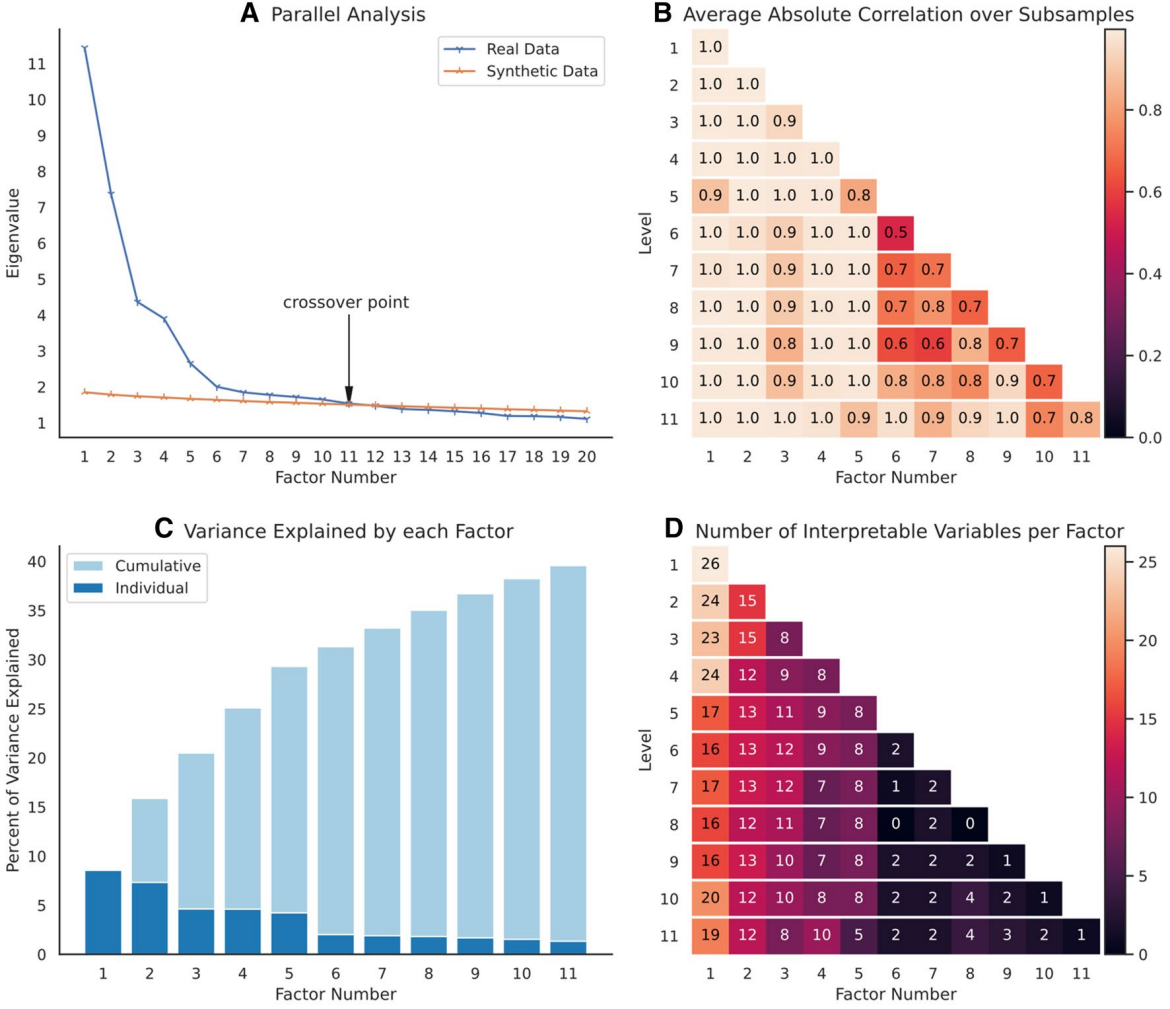
Results used to derive the maximum and optimal numbers of factors. (**A**) Parallel analysis results showing the eigenvalues by number of factors for real and synthetic data. Only the first 20 factors are shown. The number of factors before the eigenvalue of the real data became lower than that of the average of the synthetic data is marked with an arrow as the crossover point. (**B**) Factor robustness. Shown are the average absolute correlation values over subsamples. The levels of the hierarchy (i.e., the number of factors extracted in each iteration) can be found in the rows, starting from one factor at the top to eleven factors at the bottom. The factor number differs by column. Note that the order of factors might differ between levels of the hierarchy, as the factors are ordered by eigenvalue first and then made to match over subsamples. (**C**) Model fit, measured as percentage of variance explained for factor solutions with one to eleven factors (by factor in dark blue and cumulative in light blue). (**D**) Bar plot showing interpretability as the number of variables with loadings > 0.4 for each factor in each factor solution.

The parallel analysis gave an upper bound of eleven factors to how many factors should be considered, as this was the number of factors for which the squared loadings for synthetic data and the real dataset crossed over (see Fig. 1A).

*Robustness* results (Fig. 1B) from the subsampling procedure showed that from level six onwards, additional factors had low absolute correlation values, while the first five factors remained relatively stable. Four factors gave almost perfect robustness with average absolute correlation values of ~ 1.0 for every factor. With five factors, two of them exhibited lower robustness (0.8 and 0.9).

The *model* *fit* (Fig. 1C), as measured by the percent of variance explained by each factor, leveled off after five factors, explaining 29.3% of variance. Finally, in terms of *interpretability*, solutions with six factors and more only added factors with few interpretable variables (Fig. 1D).

Taken together, these results suggested a factor dimensionality of four to five factors. Five factors gave the highest model fit, while still being interpretable and relatively robust. Four factors yielded better robustness at the expense of less explained variance. Consequently, both the four- and five-factor solutions were considered for interpretation.

### Dimensionality of the clustering space

**Figure 2:**
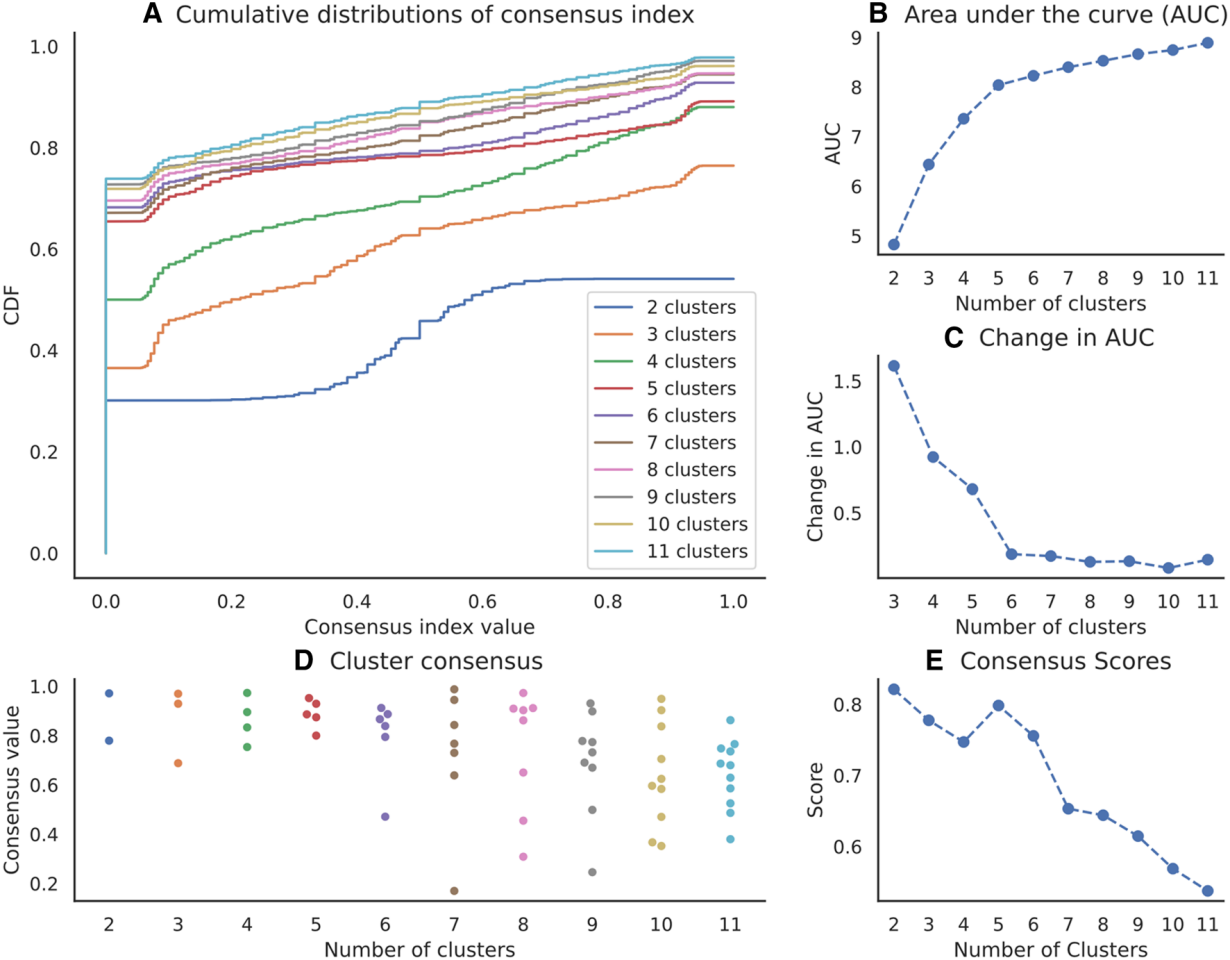
Consensus clustering. (**A**) Cumulative density functions (CDFs) of the consensus index. (**B**) Area under the curve (AUC) for the CDF of each number of clusters. (**C**) Change in AUC going to the number of clusters on the x-axis. (**D**) Cluster consensus values for each cluster by number of clusters. (**E**) Consensus score by number of clusters. For more detailed explanations on each measure, see text.

The cumulative density functions (CDFs) of the consensus index show the fraction of consensus indices below a given value (Fig. 2A). Recall that the consensus index for a given pair of items quantifies how often both items were clustered together when drawn in the same subsample. For perfect consensus, all the consensus indices would either be 1.0 (always in the same cluster) or 0 (never in the same cluster), resulting in a step function for the CDF. Consequently, the closer the CDF to a step function, the better the consensus. Upon visual inspection, the five-cluster CDF resembled a step shape the most.

Further investigations confirmed five clusters as an optimal solution. Indeed, the CDF’s area under the curve (AUC) leveled off from five clusters onwards (Fig. 2B), and the change in AUC became negligible (Fig. 2C). Furthermore, for five clusters, cluster-wise consensus values were homogeneously high (Fig. 2D) and the consensus score clearly peaked (Fig. 2E). Taken together, we thus set on 5 clusters as an optimal clustering solution.

### Interpretation of the latent space

To be useful in a predictive setting, the extracted latent variables need to have a meaningful interpretation. Here, we offer our interpretation of these variables. Note that the sign of the loadings is a consequence from the rotation used and is thus arbitrary. Where stated, the polarity of the loadings was reversed to allow for a more intuitive interpretation.

#### Four-factor solution (Fig. 3)

**Figure 3:**
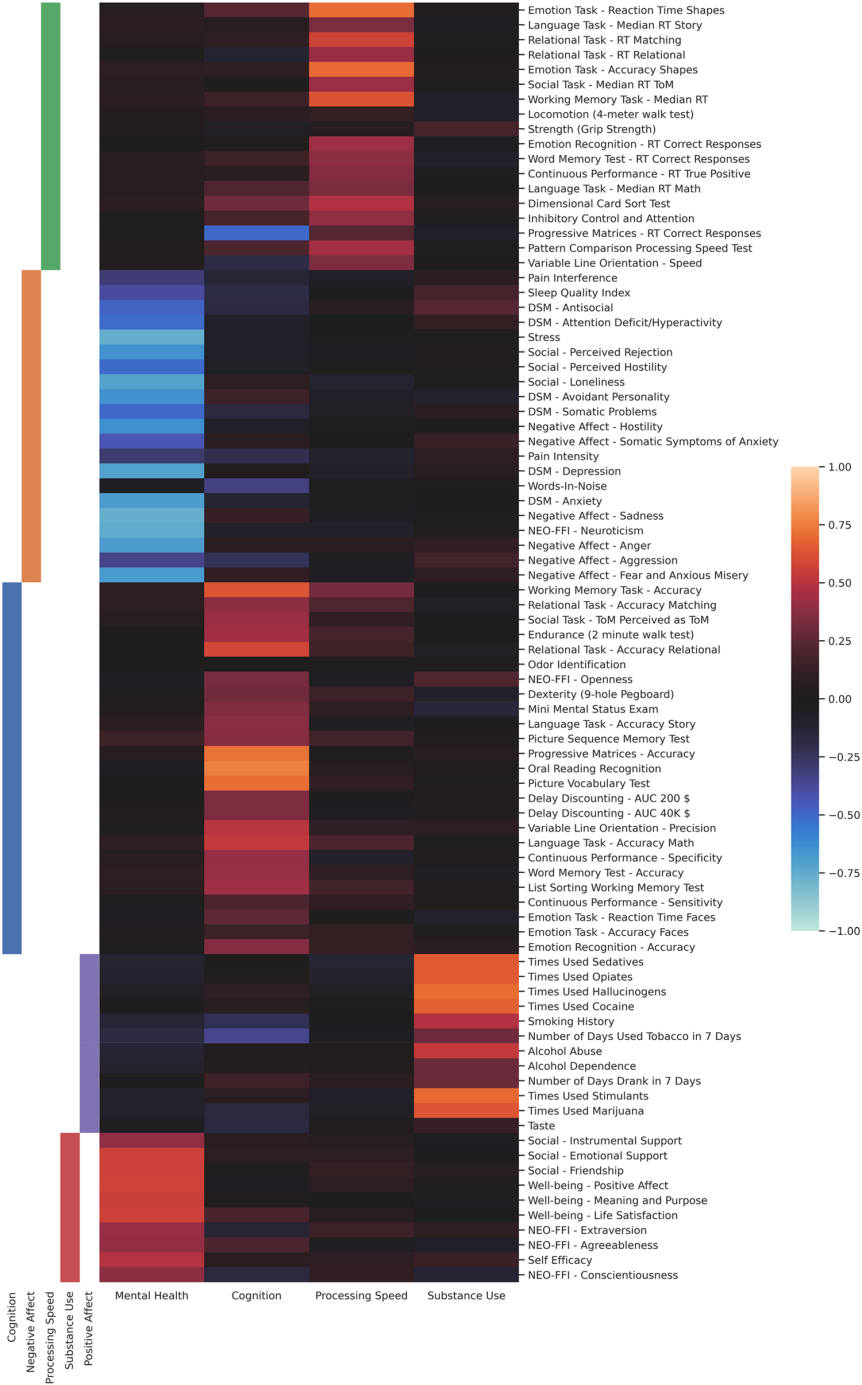
Loading matrix of the four-factor solution with the clustering of the five-cluster consensus matrix on the left, using the discovery data. The rows of the matrix are reordered using the clusters derived from consensus clustering. The factors are ordered by their explained variance as columns, decreasing from left to right.

The first factor loaded positively on well-being items such as *Positive* *Affect*, *Meaning* *and* *Purpose*, and *Life* *Satisfaction*, on social items as *Friendship*, *Emotional* *Support*, and *Instrumental* *Support*, and on the personality traits *Conscientiousness*, *Extraversion*, and *Agreeableness*, as well as on *Self-Efficacy*. There were also strong negative loadings on items related to mental illness (DSM items) such as *Depression*, *Anxiety*, *Avoidant* *Personality*, and to a lesser degree *Attention* *Deficit/Hyperactivity*, *Somatic* *problems*, and *Antisocial* *Personality*. Additionally, variables on negative affect (*Anger,* *Fear* *and* *Anxious* *Misery,* *Sadness,* *Hostility,* *Somatic* *Problems* *of* *Anxiety,* *Aggression*), social issues (*Perceived* *Hostility,* *Perceived* *Rejection,* *Loneliness*), as well as *Stress,* *Neuroticism,* and *Sleep* *Quality* loaded negatively on this factor. Taken together, this factor was thus interpreted as reflecting Mental Health.

The second factor displayed positive loadings for the accuracy of various cognitive tasks (most notably *Progressive* *Matrices,* *Oral* *Reading* *Recognition,* *Picture* *Vocabulary* *Test,* *Working* *Memory* *Task,* *Language* *Task,* *Relational* *Task,* and *Variable* *Line* *Orientation*). Additionally, it was related to the personality trait *Openness*, as well as *Endurance* and *Dexterity*. There were negative loadings on the reaction time of the *Progressive* *Matrices* task, *Words* *in* *Noise* and *Number* *of* *Days* *Used* *Tobacco* *in* *7* *Days*. As the variables were not limited to a specific cognitive domain, but instead encompassed memory, fluid intelligence, executive function, language, and others, we decided to dub this factor Cognition.

Factor number three loaded strongly on various reaction time measures (e.g., of *Variable* *Line* *Orientation,* *Relational* *Task,* *Working* *Memory,* *Emotion* *Task,* *Word* *Memory* *Test,* *Language* *Task*) and on cognitive tasks that require quick processing of stimuli, such as the *Dimensional* *Card* *Sort* *Test,* *Inhibitory* *Control* *and* *Attention,* and the *Pattern* *Comparison* *Processing* *Speed* *Test*. Consequently, we named this factor Processing Speed. The only result not fully in line with this interpretation was the strong loading on the accuracy for shapes in the *Emotion* *Task*.

The fourth factor loaded on various substance use items (times used *Opiates,* *Sedatives,* *Cocaine,* *Hallucinogens,* *Stimulants,* *Marijuana*), *Smoking* *History* and *Alcohol* *Abuse* (but not as much on *Alcohol* *Dependence* or *Number* *of* *Days* *Drank* *in* *7* *Days*). We thus interpreted it as reflective of Substance Use.

#### Five-factor solution (Fig. 4)

**Figure 4:**
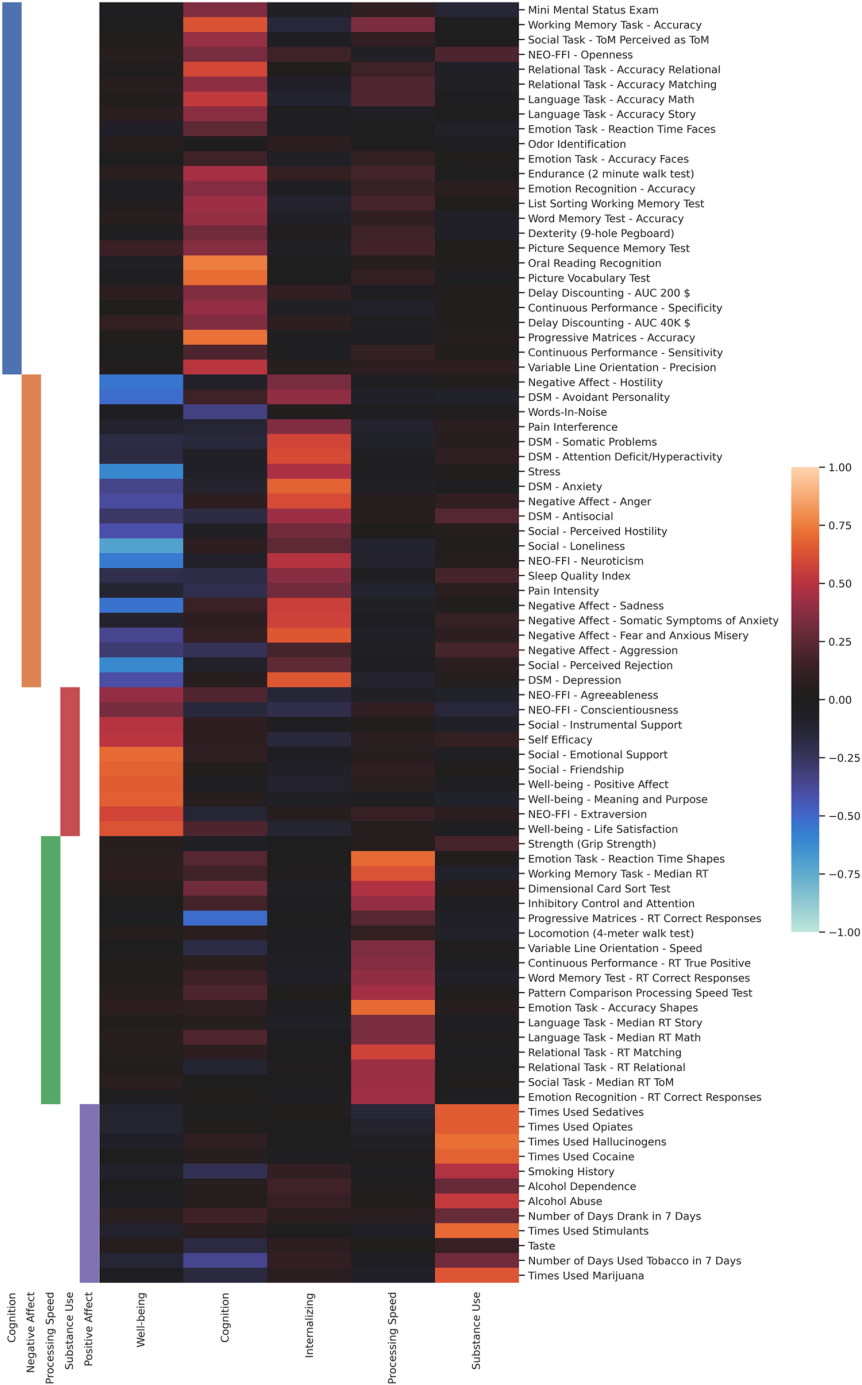
Loading matrix of the five-factor solution with the clustering of the five-cluster consensus matrix on the left, using the discovery data. The rows of the matrix are reordered using the clusters derived from consensus clustering. The factors are ordered by their explained variance as columns, decreasing from left to right.

With five factors, the factors for *Cognition,* *Processing Speed* and *Substance Use* retained very similar patterns. The first factor showed a slightly different loading pattern compared to the *Mental Health* factor of the four-factor solution: there were high loadings on social items such as *Emotional* *Support,* *Friendship,* and *Instrumental* *Support,* on the well-being related items *Meaning* *and* *Purpose,* *Positive* *Affect,* and *Life* *Satisfaction,* and on the personality traits *Extraversion,* *Agreeableness,* and *Conscientiousness*. There were also negative loadings for the social items *Loneliness,* *Perceived* *Hostility,* and *Perceived* *Rejection,* and for the affect-related variables *Hostility,* *Sadness*, *Anger,* and *Fear* *and* *Anxious* *Misery*. Additionally, this factor loaded negatively on *Stress,* the personality trait *Neuroticism*, as well as the DSM items *Depression,* *Avoidant* *Personality* and *Anxiety*. We decided to call it *Well-Being*.

The third factor was characterized by high loadings on DSM items, in particular *Anxiety,* *Somatic* *Problems,* *Attention* *Deficit/Hyperactivity,* *Depression,* and *Avoidant* *Personality.* Furthermore, it loaded high on the negative affect variables *Anger,* *Sadness,* *Fear* *and* *Anxious* *Misery,* and *Somatic* *Symptoms* *of* *Anxiety.* Finally, there were also high loadings on *Stress,* and *Neuroticism,* and moderate loadings on *Perceived* *Hostility,* *Perceived* *Rejection,* and *Hostility.* We thus called this factor Internalizing, to emphasize that this factor was driven mainly by items related to inwardly directed negative mood.

#### Factor hierarchy

**Figure 5:**
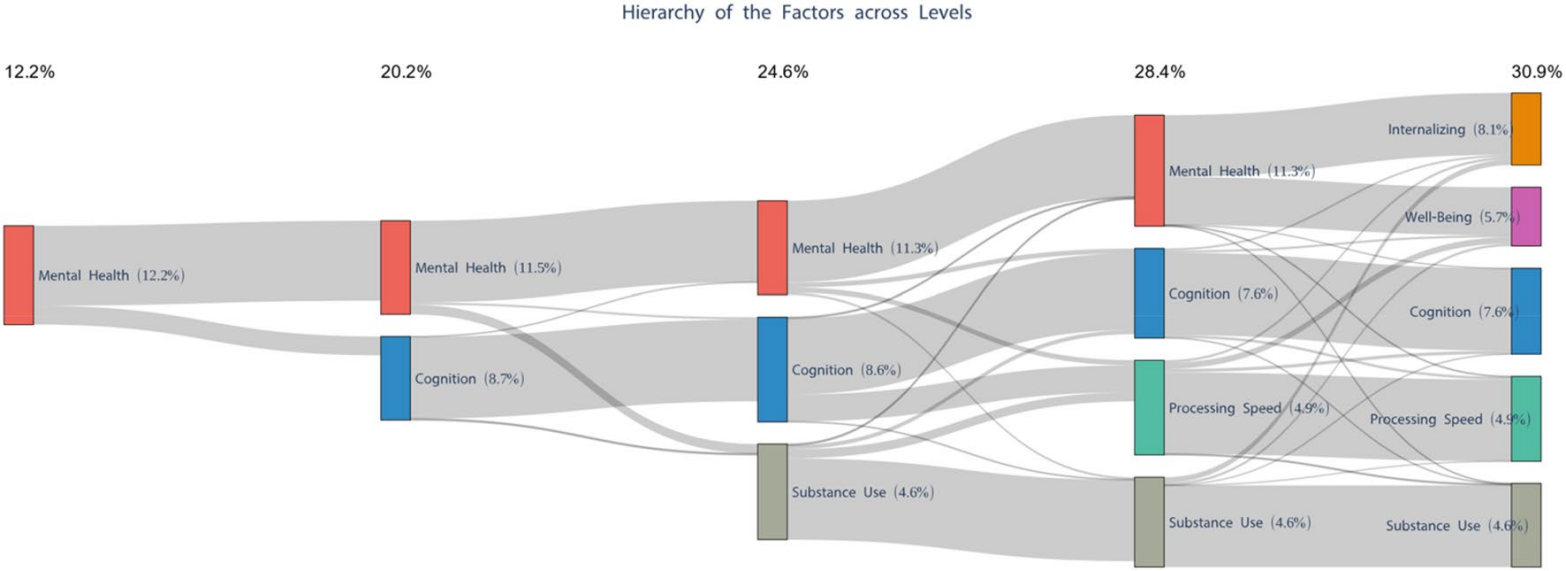
Sankey plot showing the flow of variance throughout the factor hierarchy. The absolute correlation value is plotted as the width of the bar. Percentage of variance explained is shown in brackets next to the name of the factor, and the total variance explained for a given number of factors at the top. Notice how Cognition splits in two separate factors respectively reflective of accuracy and speed, and how Mental Health splits into Well-Being and Internalizing. Names of the factors were derived from interpreting the respective factor loading matrices.

To further relate the above latent representations, we extracted a hierarchy of factors across factor solutions (Fig. 5). The width of the bars that flow into a factor show how much of its variance was already explained by the previous solution. Mental Health and Cognition were the first two extracted factors, and Substance Use was then retrieved from three factors onwards, largely accounting for previously unaccounted variance. Processing Speed appeared with four factors, partly displaying variance that was previously included in Cognition. Eventually, with five factors, Mental Health was neatly split into Well-Being and Internalizing.

#### Factors and consensus clustering

As can be seen from Figs. 3 and 4, clustering results lined up quite well with the factor loading patterns of Cognition, Processing Speed and Substance Use, which is why they were named accordingly. The clustering differed from what would be expected from the loading pattern of the items that load highly on the Mental Health factor. Instead of belonging to one cluster comprised of items with high absolute loadings on this factor, there are two clusters. One cluster contains items with positive valence (e.g. *Emotional* *Support*, *Meaning* *and* *Purpose*), while the other contains items with negative valence (e.g. *Loneliness*, *Stress*). Hence, they were named Positive Affect and Negative Affect, respectively.

### Replication of the latent structure

The replication dataset was used to establish which of the factors found in the discovery data would replicate in an independent dataset using the same variables. To this aim, EFA using the same parameters as on the discovery data was used to extract four or five factors from the replication set. Similarly, the clustering outcomes with five clusters were compared between sub-datasets. Additionally, CFA was employed to statistically assess the quality of the two different factor solutions.

To establish if this factor structure could be replicated on a completely different dataset with variables from similar domains, but captured using different tests and questionnaires, we repeated the EFA on the UCLA dataset.

#### Similarity of factors

Figures S3 and S4 show the loadings and cluster assignments of the replication data. The similarity of factors between the two sub-datasets is quantified using Pearson’s correlation, as shown in Figure S5. While there is good correspondence for all four factors in the four-factor solution, the pattern is different for five factors. Here, Internalizing does not have a direct counterpart in the replication data. Instead, a factor with high loadings from all questionnaires querying aspects of social life emerges, which we called Social Support.

#### Similarity of clusters

The clustering shows almost perfect overlap between the two sub-datasets. Only the variables *Strength* *(Grip* *Strength)*, *Words-In-Noise*, and *Odor* *identification* differ in their allocation to clusters in the replication data. The similarity between clustering solutions is also reflected in the adjusted Rand index (0.92) and the adjusted mutual information (0.91), for both of which the maximum possible value is 1.

#### Confirmatory factor analysis

Table 1 depicts the fit indices of the four- and five-factor models of CFA. The model based on the HCP variable categories did not converge. The five-factor model showed a better fit than the four-factor model for all fit indices. The indices drew a mixed picture in terms of how well the models could fit the data. The incremental fit indices (CFI and TLI) were below the recommended cutoff of 0.95^34^ for both models. However, the 90% confidence interval of the five-factor model for RMSEA was in the adequate range (< 0.08^35^), and the SRMR further supported the models, with both values below the recommended cutoff of 0.08^34^.

**Table 1:** Fit index values.

Model	Chi-Square (df)	CFI	TLI	RMSEA (90% CI)	SRMR
4-factor model	4065.75 (813)	0.739	0.731	0.081 (0.079–0.084)	0.068
5-factor model	3665.38 (847)	0.775	0.761	0.074 (0.072–0.077)	0.064

*df* degrees of freedom*,* *CFI* Comparative Fit Index*,* *TLI* Tucker Lewis Index*,* *RMSEA,* Root Mean Square Error of Approximation*,* *Cl* Confidence Interval*,* *SRMR* standardized root mean square residual*.*

#### EFA of the UCLA data

Figures S6 and S7 show the results of the EFA using the UCLA dataset. First, we ran the EFA using all variables fulfilling our criteria (see “Methods”), the six-factor solution of which is shown in Figure S6. Here, the first three factors were similar in their content to the factors of the HCP four-factor solution. The first factor had strong loadings from items related to impulsiveness, anxiety, depression, and other constructs related to mental health, which could thus be equivalent to the Mental Health factor from the HCP data. The second factor showed high loadings from neuropsychological assessments and neurocognitive tasks, being similar in content to the Cognition factor in the HCP data. Third, the reaction time variables combined into a factor akin to the Processing Speed factor from before. The last three factors mirrored different aspects of substance use. Unlike in the HCP data, there was a strong separation between alcohol consumption and alcohol dependence (factor 4), smoking (factor 5), and alcohol abuse (factor 6).We suspected that this separation might have happened due to low variation in the substance use items, which were coded as 0, 1, and 2, with very few subjects scoring as 2. Thus, the variation in the data might not have been enough to reveal any meaningful correlations between these variables based on which the EFA would be able to extract factors. Consequently, we repeated the EFA without these variables, the results of which can be seen in Figure S7. In accordance with our hypothesis, the three-factor solution here showed factors with similar loadings as in the first three factors before, which we deemed equivalent to the HCP factors Mental Health, Cognition, and Processing Speed.

## Discussion

In this study, we explored the latent structure of the HCP behavioral data to find factors that are *robust,* *interpretable* and show good *model* *fit.* According to the chosen criteria, we found a four-factor solution with the domains Mental Health, Cognition, Processing Speed, Substance Use, and a five-factor solution with factors for Well-Being, Cognition, Internalizing, Processing Speed, and Substance Use that explain ca. 25% and 29% of variance, respectively. These factor solutions were replicated in an independent data set with the same variables, with the four-factor solution showing higher similarity in content between datasets. Additionally, we explored the clustering of variables and how this compared to the factor structure, finding clusters similar to the four factors, except that Mental Health was separated into Positive Affect and Negative Affect clusters. It was thus shown that a discrete categorization from consensus clustering showed a similar pattern in the latent organization of behavior. Finally, we showed that similar factors as found in the HCP data could be extracted from a completely different set of variables with similar content in a dataset also including subjects with mental illness.

The only other study that, to our knowledge, explored the latent domains using HCP variables from more than one domain was performed by^17^, using Independent Component Analysis (ICA). Their behavioral dimensions were Cognition, Illicit Substance Use, Tobacco Use, Personality-Emotion, and Mental Health. It should be noted that there are differences in the set of variables used; in particular, the authors used composite measures and did not include the reaction times of the scanner tasks. The latter is likely to explain that the authors did not find a Processing Speed factor. Instead, they include a Tobacco Use component, which has a strong loading for just one item. Additionally, their solution leaves many variables unaccounted for, which have near zero loadings for all components, an issue lessened in our factoring solution. Despite these differences, there is also overlap, in that the authors also found the domains Cognition, (Illicit) Substance Use, Mental Health and Well-Being (corresponding to their Personality-Emotion domain).

An important question is how well our extracted factors and clusters correspond to other recent efforts to re-conceptualize behavior. In particular, RDoC^4^ was developed in an effort to move away from categorical classification systems of mental illness like DSM^5^, and provide a framework that allows to research mental health with more close regard to the underlying biological causes. Parallels can be drawn between RDoC’s Negative Valence Systems domain and our Internalizing and Mental Health Factors, which have high (absolute) loadings on depression- and anxiety-related items. RDoC’s Positive Valence Systems domain encompasses the constructs Reward Learning, Reward Valuation, and Reward Responsiveness, which are important mechanisms for the development of substance use disorders. Finally, the Cognitive Systems domain also contains subconstructs akin to these tested by the items with high loadings on our Cognition factor.

More recently, data-driven cognitive ontologies have been proposed. In clinical data, it has been found that a general Psychopathology factor (p-factor) offers the best summary of a wide range of symptoms of mental disorder^36^. This p-factor is comprised of subfactors Thought Disorder, Externalizing, and Internalizing, the last of which bears strong resemblance to our Internalizing factor. A similar result was found in a general population sample of adolescents, where a distress factor showed the best fit to the data^37^. This probably relates more closely to the Mental Health factor in our four-factor solution. The distress factor in their study encompassed both positive and negative items, as opposed to the Internalizing factor in the five-factor solution, for which only items with negative content showed high loadings. When interpreting these results, it should however be noted that the factor Internalizing was only found in the discovery data set.

A rather surprising finding was the emergence of a Processing Speed factor, which has so far not been described in the context of data-driven behavioral ontologies. We considered the possibility that this factor may be an artifact of the measurement method, as many of the high loadings arose from reaction time variables. Opposing this notion is the fact that there were also loadings from tasks that required quick responses, but were not reaction time measures, such as the *Card* *Sorting* and *Flanker* tasks. We take this as evidence that this factor does reflect a mental capacity rather than a method-related artifact. In fact, processing speed has been described as a fundamental feature of cognition^38^, mainly drawing from aging research, as there is considerable variation of processing speed across age^39^. It is also relevant in connection to schizophrenia. A study by^40^ suggests that the cognitive deficits as measured by IQ are due to a perturbed processing speed, a finding that has also been found in an earlier meta-analysis^41^, though the effect was mediated by medication^42^.

An important point pertains to the separability of mental health and mental illness. A critique about RDoC is that it assumes a dimensional model of psychiatric disorders, in that mental illness is viewed as the extreme end of a behavioral dimension—a conceptualization that does not work equally well for all types of disorders^43^. In contrast, the disease model presumes that there are alterations in the brain physiology that fundamentally disrupt normal behavior. The dimensional model would fit with the four-factor solution showing one positive–negative axis of mental health, in line with the results from^44^ who found such a single dimension using canonical correlation analysis. Conversely, the five-factor solution of the discovery data would be more in line with the disease model, where mental illness and normal psychological well-being constitute separate axes. The latter view has also been promoted by Keyes, who proposes a mental health dimension ranging from languishing to flourishing, which is related to but separate from mental illness^45^. It is beyond the scope of this paper to answer whether the disease or the dimensional model is more useful to conceptualize mental illness, but the points discussed above should provide some guidance regarding what to consider when deciding between the two factor solutions proposed therein. However, given that the Internalizing factor could not be reproduced in the replication data, one should probably favor using four factors, as this presents the more stable and reproducible latent structure.

Another question that could be raised is what the factor solution adds to the HCP categories. The factors differ from these categories in two important ways. First, our solution was derived in a data-driven way. Apart from the imposition of linear relationships, we do not impose any prior assumptions on the latent structure. This is fundamentally different from how psychological constructs and psychopathological taxonomies were defined historically, namely based on the insight of experts. Deriving the latent structure based on the correlational structure provides a more objective way to conceptualize the overarching categories of behavior.

Additionally, the data-driven approach offers another advantage when viewing this work with the goal of developing neuroimaging biomarkers in mind. If one were to combine all variables in a category to a sum score, every variable in that category would contribute equally to the construct of that given category. Using factor analysis enables a more nuanced view. Through the factor loadings, the contribution of each variable to a construct is quantified, such that it is reflected that some variables are more important to the construct, while others are noisier and contain less variance related to the construct at hand. This is then also taken account in the factor scores, which might thus provide a better target for prediction than simple sum scores, advancing the development of neuroimaging biomarkers of healthy and psychopathological behavior.

Second, the content of the factors differs from the HCP categories in several aspects. The categories Alertness and Motor do not emerge as factors. The Cognition category shows a speed and an accuracy component, in the factors Processing Speed and Cognition. Personality and Life Function are part of the factor Mental Health. Lastly, the Scanner Tasks align with the Cognition and Processing Speed factors, instead of forming their own.

We also explored the factor structure in a different set of variables with similar content, using the UCLA dataset. Here, three of the factors were very similar to the HCP factors Mental Health, Cognition, and Processing Speed, giving evidence that the factors found before were not specific to the HCP dataset, but can be thought of as general overarching dimensions of the more specific psychological constructs assessed in each individual questionnaire or test. This has an important implication for the development of imaging biomarkers. As it is not needed for the behavioral variables to be the same to still arrive at similar summary dimensions, datasets with very different behavioral assays can be combined to train predictive models, if one is willing to go one step up in granularity and predict these broader behavioral dimensions. A similar approach to this was already demonstrated by He and colleagues^46^, who leveraged the correlations between behavioral variables to improve the predictive accuracy of predictive models using the functional connectome. There are several avenues to build on these results. One is to explore the latent structure in a clinical sample. It could be tested if patients with mental illness show different factor scores on some dimensions, or if a different factoring is needed to best capture the latent structure in this sample. Another promising path is to examine which brain areas, components, and networks covary with each factor and if these relations correspond to what would be expected from previous research. Finally, one could test the predictability of the factor scores from brain data, similar to how it was done in^13^.

This study is not without limitations. One shortcoming is that it was not possible to test the reliability of the factor scores. While there exist re-test data for some of the subjects in the HCP data set, not all variables were measured twice. However, even though re-test reliability is often low for individual behavioral tests and questionnaires^47^, it can be high for summary measures of these^15^, making us optimistic that this may also be the case here.

Another concern lies in the interpretation of the factors, which could reflect the measurement methodology more than the putative psychological constructs. In particular, this could explain the separation between the Cognition and Processing Speed factors on one hand and Mental Health and Substance Use on the other, as one difference between these two sets of factors is that the former is driven mainly by tasks, while the latter has loadings mainly from questionnaires. This separation between tasks and questionnaires has been described recently in the context of self-regulation^15,48^. While in that case the measures were supposedly reflecting the same putative construct, this is not the case here, making it impossible to tell if the factor solution partly reflects methodology.

Finally, we want to address the finding that the Internalizing factor was not replicated in the replication cohort. While in the discovery cohort there is a separation between a healthy axis (factor Well-Being) and a pathological axis (factor Internalizing), in the replication cohort we find something akin to the Mental Health factor from the four-factor solution of the discovery data on the one hand, and a factor that combines social items on the other. While both these divisions intuitively make sense, the question remains why the Internalizing factor in particular could not be found in the replication data. One possible answer to this lies in the composition of the sample. As the HCP Young Adult data is a community sample, not many data points fall into the more pathological side of the mental health spectrum. In fact, having a mental illness was an exclusion criterion for the recruitment of subjects in this dataset^10^. Additionally, the measurement instrument used to assess differences in psychopathology^49^ was developed to distinguish different levels of symptom severity in patient, and is thus likely less precise at healthy and sub-clinical levels, i.e., having low phenotypic resolution^50^.

To conclude, this study explored the latent structure of a wide range of behavioral variables using both factor analysis and consensus clustering. Latent variables that are robust and interpretable were established. These latent variables serve as a data-driven behavioral ontology, providing a way to reduce the dimensionality of the target space in a predictive framework. We hope that in the future, the present work can help in the establishment of robust biological markers of behavior.

## Supplementary Information

Supplementary Information.

## Data Availability

Data are available through the Human Connectome Project, WU-Minn Consortium, https://www.humanconnectome.org/study/hcp-young-adult.

## Code availability

All analysis code can be found in our Github repository, including instructions on how to compute the factor scores for custom HCP samples.
