# Supplementary material for: Exploring the latent structure of behavior using the Human Connectome Project’s data
Source: Sci Rep. 2023 Jan 13;13:713. doi: 10.1038/s41598-022-27101-1 (PMC9839753; doi:10.1038/s41598-022-27101-1)
Supplement: Supplementary file 1 — Supplementary Information. [file 41598_2022_27101_MOESM1_ESM.pdf]

Supplementary Materials: Exploring the Latent Structure of Behavior Using the Human Connectome Project's Data

Mikkel Schöttner[1]*, Thomas A.W. Bolton[1,2], Jagruti Patel[1], Anjali Tarun Nahálka[1], Sandra Viera[1,3], and Patric Hagmann[1]

1 Connectomics Lab, Department of Radiology, Lausanne University Hospital and University of Lausanne (CHUV-UNIL), Switzerland

2 Neurosurgery Service and Gamma Knife Center, Lausanne University Hospital (CHUV), CH-1011 Lausanne, Switzerland

3 Department of Psychosis Studies, Institute of Psychiatry, Psychology and Neuroscience, King's College London, London, United Kingdom

*Email: mikkel.schottner@unil.ch

List of Variables Used

HCP Name	***Clear Name***
MMSE_Score	Mini Mental Status Exam
PSQI_Score	Sleep Quality Index
PicSeq_Unadj	Picture Sequence Memory Test
CardSort_Unadj	Dimensional Card Sort Test
Flanker_Unadj	Inhibitory Control and Attention
PMAT24_A_CR	Progressive Matrices - Accuracy
PMAT24_A_RTCR	Progressive Matrices - RT Correct Responses
ReadEng_Unadj	Oral Reading Recognition
PicVocab_Unadj	Picture Vocabulary Test
ProcSpeed_Unadj	Pattern Comparison Processing Speed Test
DDisc_AUC_200	Delay Discounting - AUC 200 $
DDisc_AUC_40K	Delay Discounting - AUC 40K $
VSPLOT_CRTE	Variable Line Orientation - Speed
VSPLOT_OFF	Variable Line Orientation - Precision
SCPT_SEN	Continuous Performance - Sensitivity
SCPT_SPEC	Continuous Performance - Specificity
SCPT_TPRT	Continuous Performance - RT True Positive
IWRD_TOT	Word Memory Test - Accuracy
IWRD_RTC	Word Memory Test - RT Correct Responses
ListSort_Unadj	List Sorting Working Memory Test
ER40_CR	Emotion Recognition - Accuracy
ER40_CRT	Emotion Recognition - RT Correct Responses
AngAffect_Unadj	Negative Affect - Anger
AngHostil_Unadj	Negative Affect - Hostility
AngAggr_Unadj	Negative Affect - Aggression
FearAffect_Unadj	Negative Affect - Fear and Anxious Misery
FearSomat_Unadj	Negative Affect - Somatic Symptoms of Anxiety
Sadness_Unadj	Negative Affect - Sadness
LifeSatisf_Unadj	Well-being - Life Satisfaction
MeanPurp_Unadj	Well-being - Meaning and Purpose
PosAffect_Unadj	Well-being - Positive Affect
Friendship_Unadj	Social - Friendship
Loneliness_Unadj	Social - Loneliness
PercHostil_Unadj	Social - Perceived Hostility
PercReject_Unadj	Social - Perceived Rejection
EmotSupp_Unadj	Social - Emotional Support

InstruSupp_Unadj	Social - Instrumental Support
PercStress_Unadj	Stress
SelfEff_Unadj	Self Efficacy
Emotion_Task_Face_Acc	Emotion Task - Accuracy Faces
Emotion_Task_Face_Median_RT	Emotion Task - Accuracy Shapes
Emotion_Task_Shape_Acc	Emotion Task - Reaction Time Faces
Emotion_Task_Shape_Median_RT	Emotion Task - Reaction Time Shapes
Language_Task_Story_Acc	Language Task - Accuracy Story
Language_Task_Story_Median_RT	Language Task - Median RT Story
Language_Task_Math_Acc	Language Task - Accuracy Math
Language_Task_Math_Median_RT	Language Task - Median RT Math
Relational_Task_Match_Acc	Relational Task - Accuracy Matching
Relational_Task_Match_Median_RT	Relational Task - RT Matching
Relational_Task_Rel_Acc	Relational Task - Accuracy Relational
Relational_Task_Rel_Median_RT	Relational Task - RT Relational
Social_Task_TOM_Perc_TOM	Social Task - ToM Perceived as ToM
Social_Task_TOM_Median_RT_TOM	Social Task - Median RT ToM
WM_Task_Acc	Working Memory Task - Accuracy
WM_Task_Median_RT	Working Memory Task - Median RT
Endurance_Unadj	Endurance (2-minute walk test)
GaitSpeed_Comp	Locomotion (4-meter walk test)
Dexterity_Unadj	Dexterity (9-hole Pegboard)
Strength_Unadj	Strength (Grip Strength)
NEOFAC_A	NEO-FFI - Agreeableness
NEOFAC_O	NEO-FFI - Openness
NEOFAC_C	NEO-FFI - Conscientiousness
NEOFAC_N	NEO-FFI - Neuroticism
NEOFAC_E	NEO-FFI - Extraversion
Noise_Comp	Words-In-Noise
Odor_Unadj	Odor Identification
PainIntens_RawScore	Pain Intensity
PainInterf_Tscore	Pain Interference
Taste_Unadj	Taste
DSM_Depr_Raw	DSM - Depression
DSM_Anxi_Raw	DSM - Anxiety
DSM_Somp_Raw	DSM - Somatic Problems
DSM_Avoid_Raw	DSM - Avoidant Personality
DSM_Adh_Raw	DSM - Attention Deficit/Hyperactivity
DSM_Antis_Raw	DSM - Antisocial Personality
Num_Days_Drank_7days	Number of Days Drank in 7 Days
SSAGA_Alc_D4_Dp_Sx	Alcohol Dependence
SSAGA_Alc_D4_Ab_Sx	Alcohol Abuse
Num_Days_Used_Any_Tobacco_7days	Number of Days Used Tobacco in 7 Days
SSAGA_TB_Smoking_History	Smoking History
SSAGA_Times_Used_Cocaine	Times Used Cocaine
SSAGA_Times_Used_Hallucinogens	Times Used Hallucinogens
SSAGA_Times_Used_Opiates	Times Used Opiates
SSAGA_Times_Used_Sedatives	Times Used Sedatives
SSAGA_Times_Used_Stimulants	Times Used Stimulants
SSAGA_Mj_Times_Used	Times Used Marijuana

Percent of Variance Explained

1 factor	8.551763
2 factors	15.870733
3 factors	20.488851
4 factors	25.085399
5 factors	29.307948
6 factors	31.316349
7 factors	33.213306
8 factors	35.025679
9 factors	36.706063
10 factors	38.234298
11 factors	39.567732

Variables used in the CFA

Four factors

Factor 1:

AngAffect_Unadj,

AngHostil_Unadj,

FearAffect_Unadj,

Sadness_Unadj,

LifeSatisf_Unadj,

MeanPurp_Unadj,

PosAffect_Unadj,

Friendship_Unadj,

Loneliness_Unadj,

PercHostil_Unadj,

PercReject_Unadj,

EmotSupp_Unadj,

PercStress_Unadj,

SelfEff_Unadj,

NEOFAC_N,

DSM_Depr_Raw,

DSM_Anxi_Raw,

DSM_Somp_Raw,

DSM_Avoid_Raw,

DSM_Adh_Raw,

DSM_Antis_Raw

Factor 2:

PMAT24_A_CR,

PMAT24_A_RTCR,

ReadEng_Unadj,

PicVocab_Unadj,
VSPLOT_OFF,
Language_Task_Math_Acc,
Relational_Task_Rel_Acc,
WM_Task_Acc

Factor 3:
CardSort_Unadj,
Emotion_Task_Face_Median_RT,
Emotion_Task_Shape_Median_RT,
Relational_Task_Match_Median_RT,
WM_Task_Median_RT

Factor 4:
SSAGA_Alc_D4_Ab_Sx,
SSAGA_TB_Smoking_History,
SSAGA_Times_Used_Cocaine,
SSAGA_Times_Used_Hallucinogens,
SSAGA_Times_Used_Opiates,
SSAGA_Times_Used_Sedatives,
SSAGA_Times_Used_Stimulants,
SSAGA_Mj_Times_Used

Five Factors

Factor 1:
AngHostil_Unadj,
Sadness_Unadj,
LifeSatisf_Unadj,
MeanPurp_Unadj,
PosAffect_Unadj,
Friendship_Unadj,
Loneliness_Unadj,
PercReject_Unadj,
EmotSupp_Unadj,
InstruSupp_Unadj,
PercStress_Unadj,
SelfEff_Unadj,
NEOFAC_N,
NEOFAC_E,
DSM_Avoid_Raw],

Factor 2:

PMAT24_A_CR,
PMAT24_A_RTCR,
ReadEng_Unadj,
PicVocab_Unadj,
VSPLOT_OFF,
Language_Task_Math_Acc,
Relational_Task_Rel_Acc,
WM_Task_Acc

Factor 3:

AngAffect_Unadj,
FearAffect_Unadj,
FearSomat_Unadj,
Sadness_Unadj,
PercStress_Unadj,
NEOFAC_N,
DSM_Depr_Raw,
DSM_Anxi_Raw,
DSM_Somp_Raw,
DSM_Adh_Raw

Factor 4:

CardSort_Unadj,
Emotion_Task_Face_Median_RT,
Emotion_Task_Shape_Median_RT,
Relational_Task_Match_Median_RT,
WM_Task_Median_RT

Factor 5:

SSAGA_Alc_D4_Ab_Sx,
SSAGA_TB_Smoking_History,
SSAGA_Times_Used_Cocaine,
SSAGA_Times_Used_Hallucinogens,
SSAGA_Times_Used_Opiates,
SSAGA_Times_Used_Sedatives,
SSAGA_Times_Used_Stimulants,
SSAGA_Mj_Times_Used

Supplementary Figures

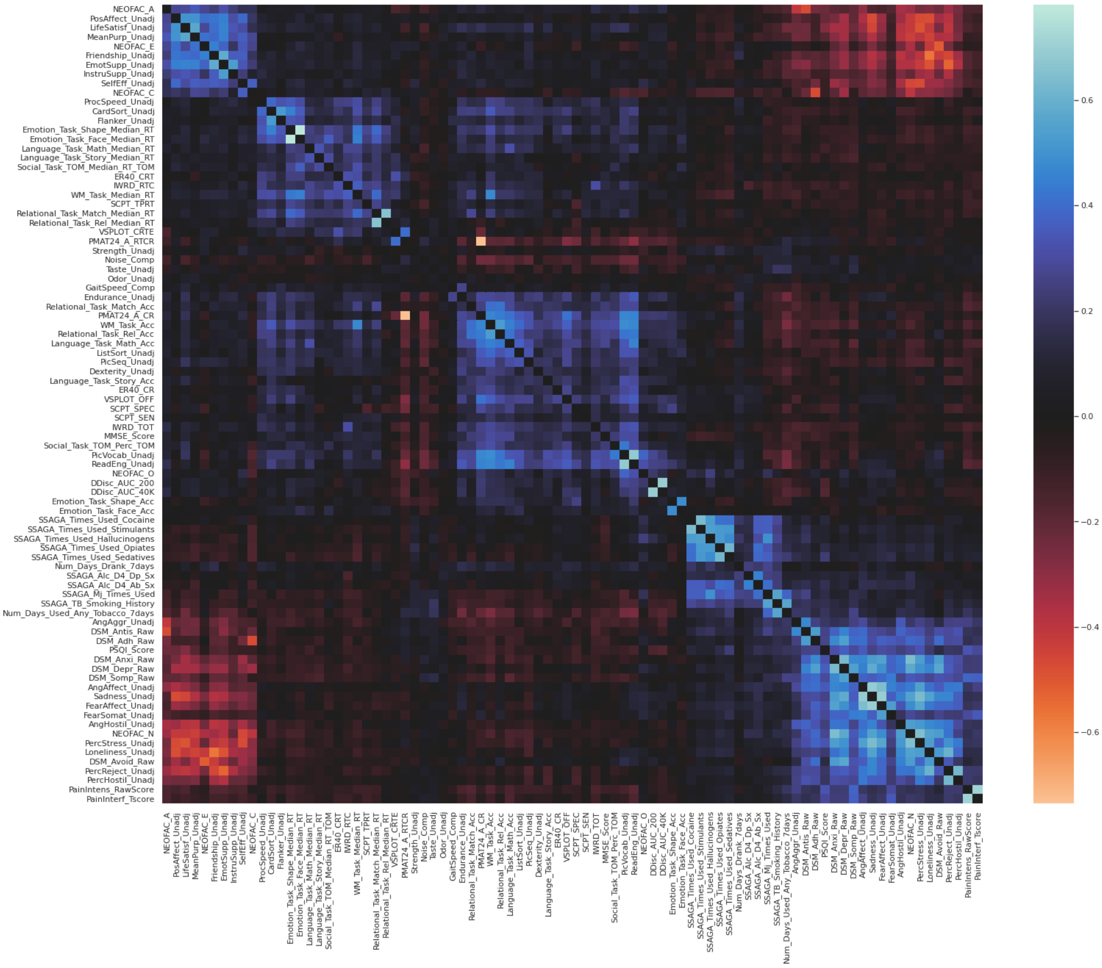

Figure S1: Correlation matrix of the behavioral variables, ordered according to the optimal leaf order derived via hierarchical clustering.

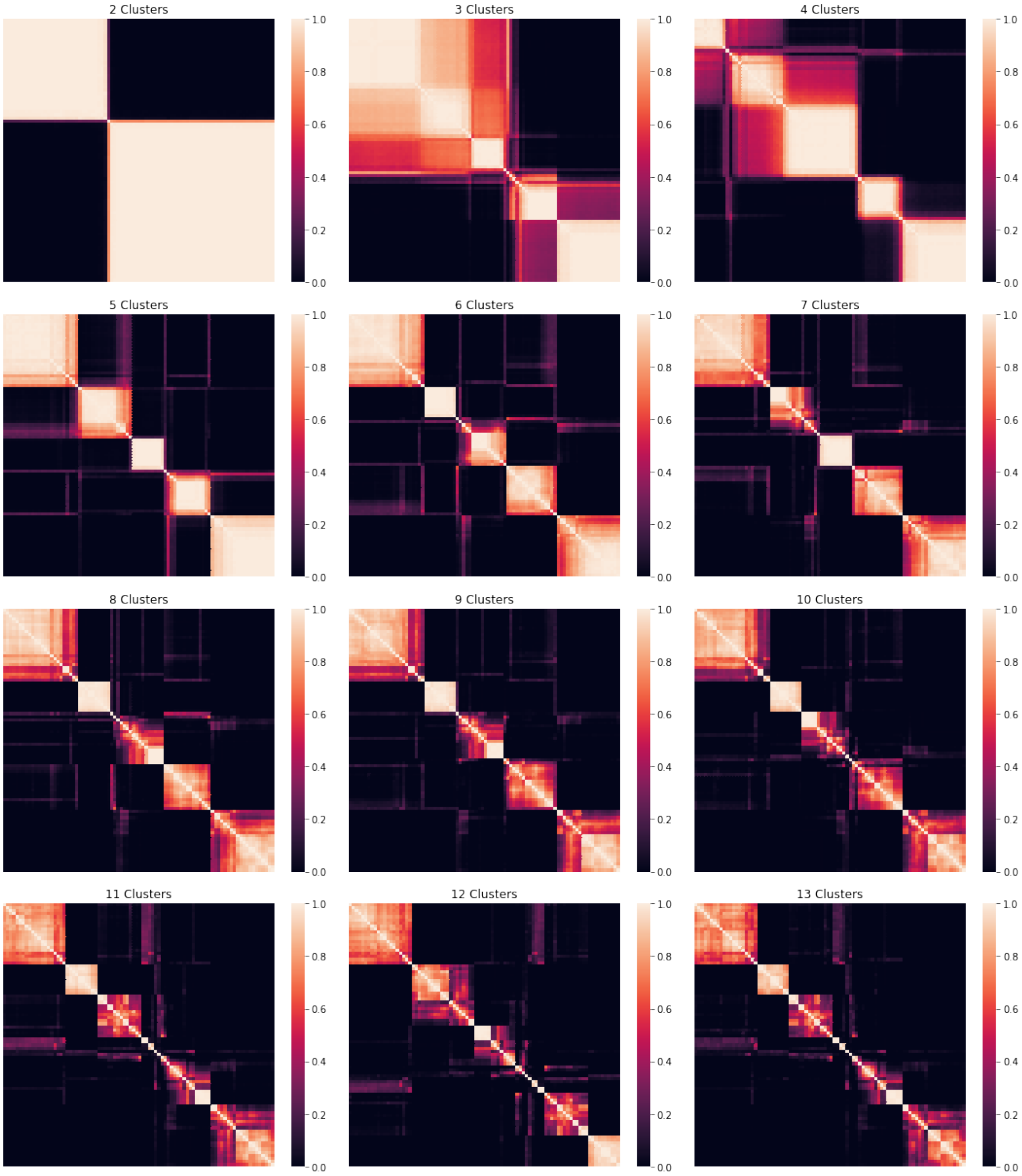

Figure S2: Consensus matrices for the consensus clustering. Rows and columns are reordered using hierarchical clustering.

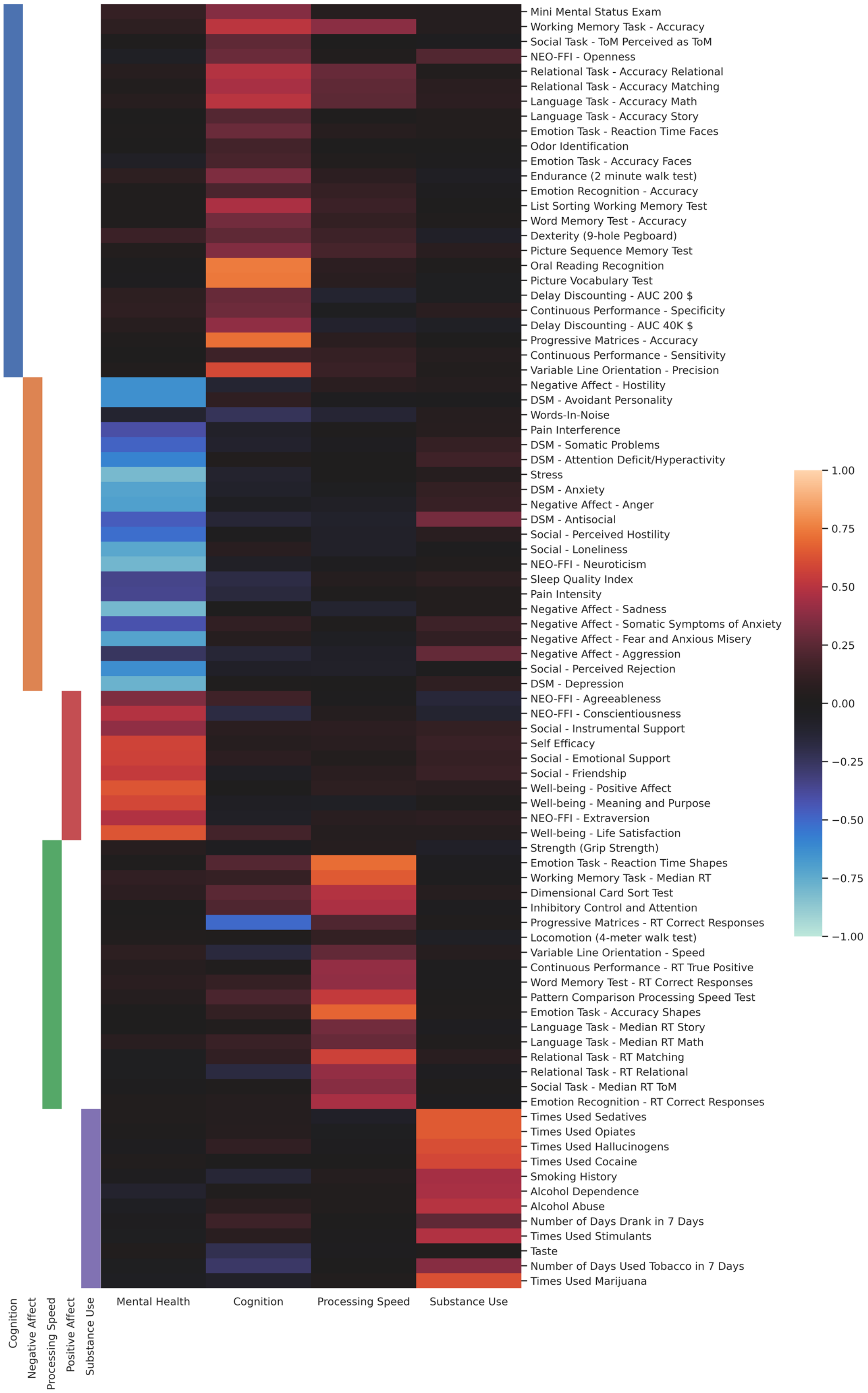

Figure S3: Loading matrix of the four-factor solution with the clustering of the five-cluster consensus matrix on the left, using the replication data. The rows of the matrix are reordered using the clusters derived from consensus clustering. The factors are ordered by their explained variance as columns, decreasing from left to right.

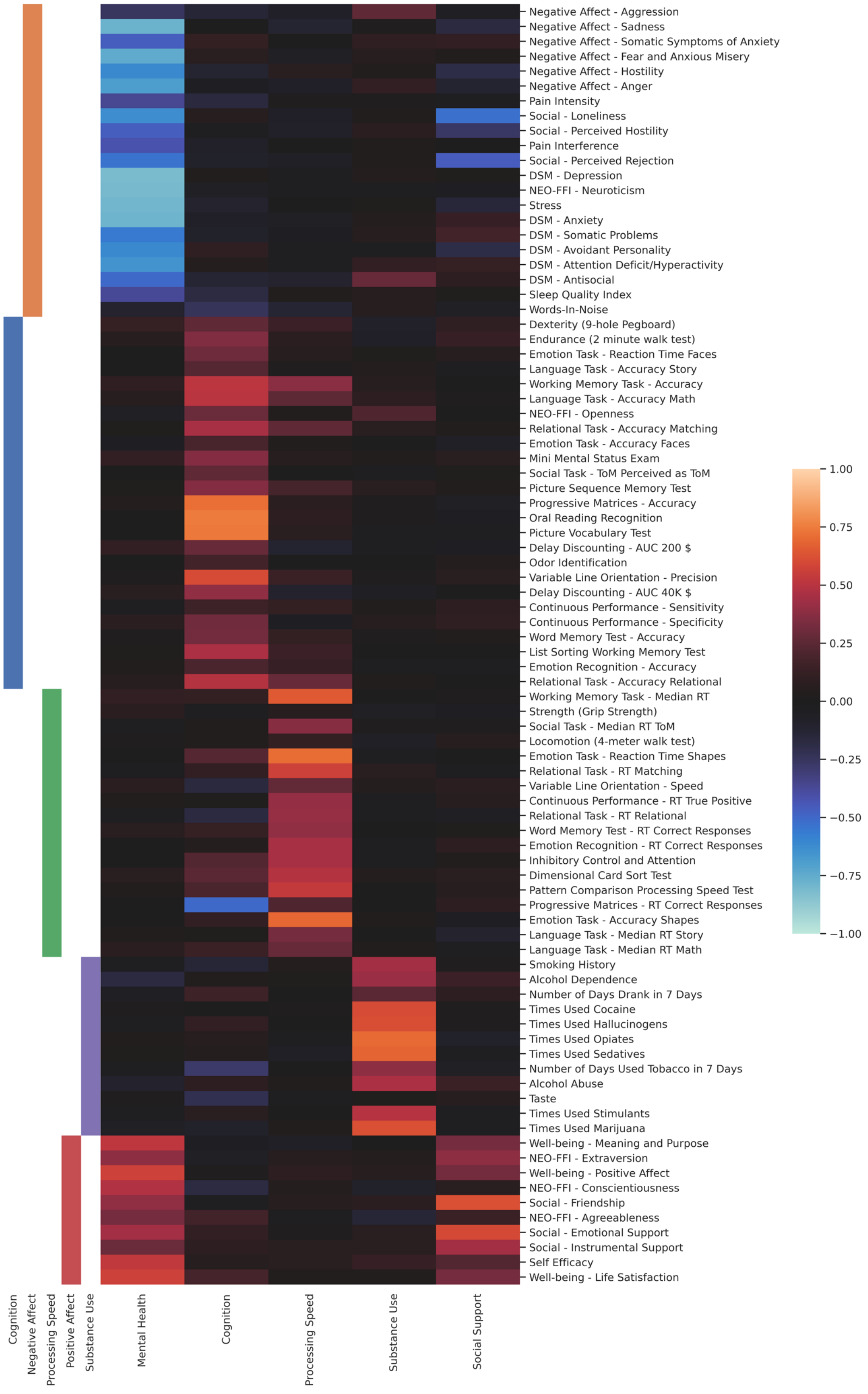

Figure S4: Loading matrix of the five-factor solution with the clustering of the five-cluster consensus matrix on the left, using the replication data. The rows of the matrix are reordered using the clusters derived from consensus clustering. The factors are ordered by their explained variance as columns, decreasing from left to right.

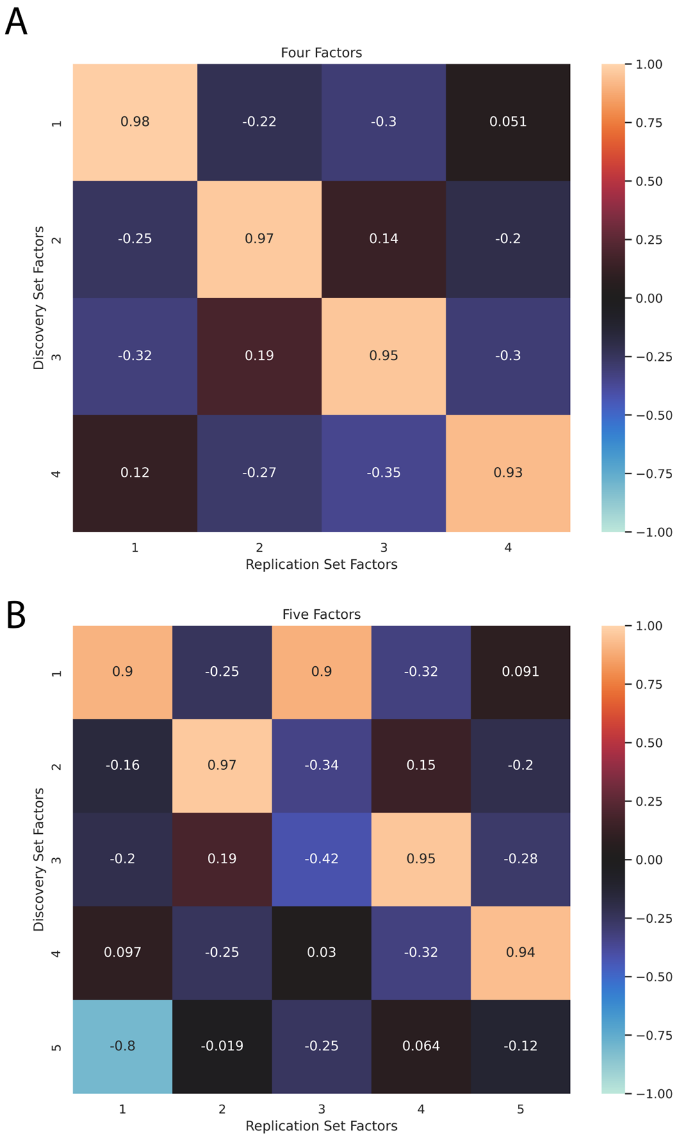

Figure S5: Correlation matrix quantifying the similarity between the factor loadings of discovery and replication set. Shown are the similarities of the four-factor solution (A) and the five-factor solution (B) as quantified by Pearson's correlation coefficient.

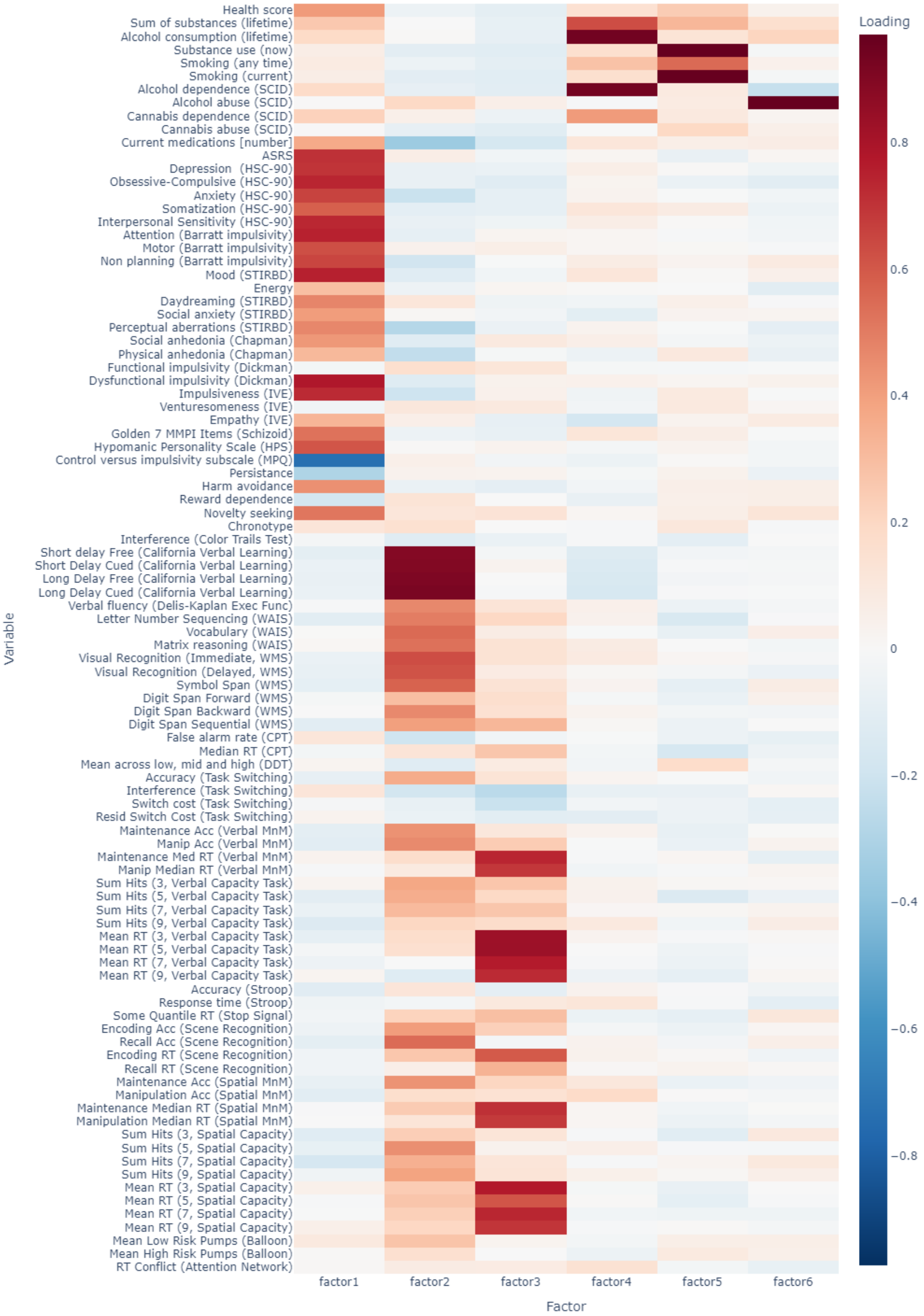

Figure S6: Loading matrix of the six-factor solution of the UCLA data. The factors are ordered by their explained variance as columns, decreasing from left to right, the variables correspond to the rows of the matrix.

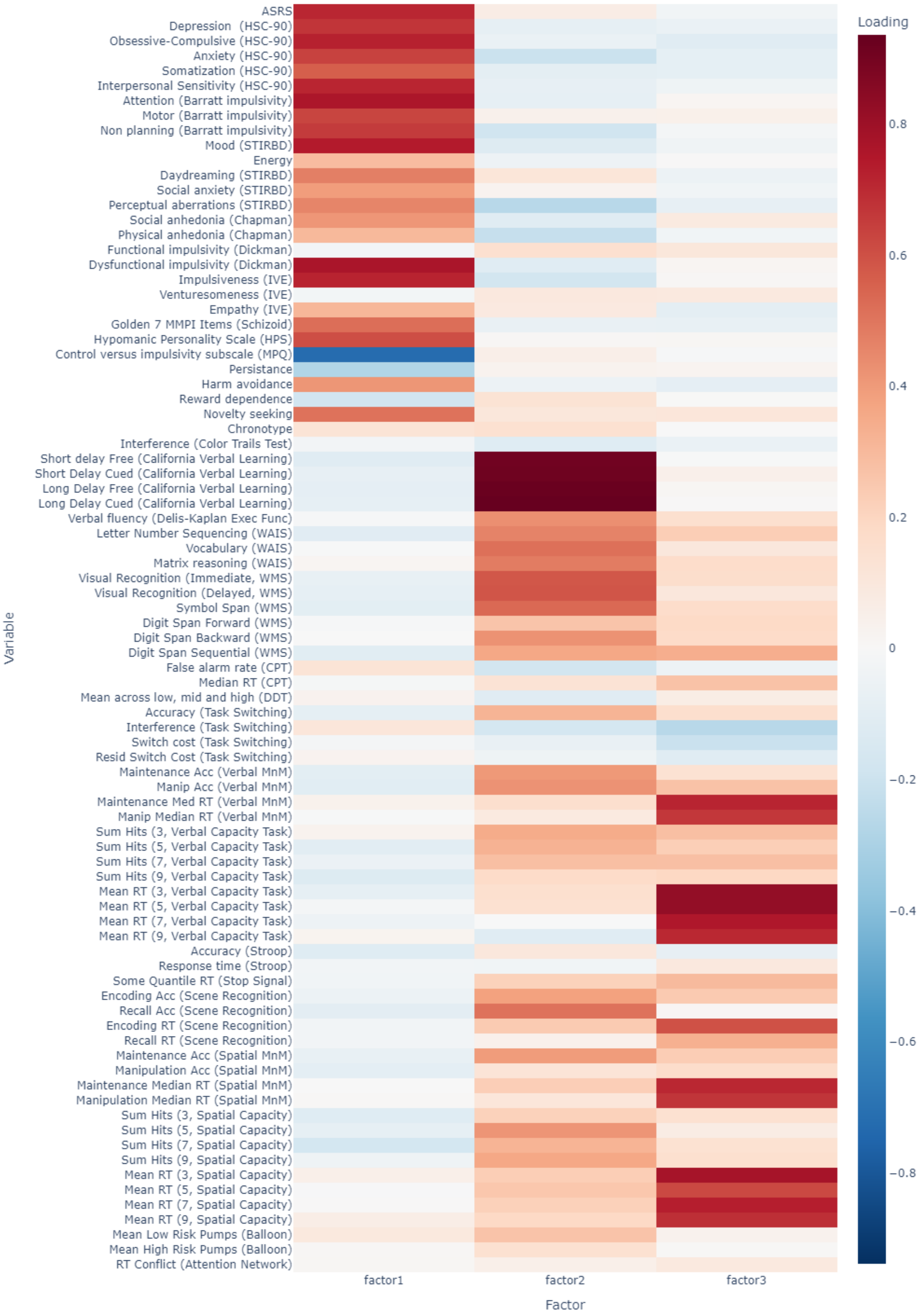

Figure S7: Loading matrix of the three-factor solution of the UCLA data, excluding the substance use items. The factors are ordered by their explained variance as columns, decreasing from left to right, the variables correspond to the rows of the matrix.
